# Some crucial questions to be answered about abandoned embryos

**DOI:** 10.5935/1518-0557.20180077

**Published:** 2019

**Authors:** Vera Lúcia Raposo

**Affiliations:** 1 Faculty of Law, University of Macao, Macao, China

The drama of abandoned embryos is present all around the world. People use reproductive techniques to generate in vitro embryos, but when they are not immediately transferred to a uterus they become abandoned embryos and their existence may remain in stand by for years, even decades.

In order to avoid surplus embryos to be abandoned the law must clarify some crucial questions:

i) Should these embryos be treated as a human biological material (i.e., *res* ) or as children in vitro (i.e., persons) (Raposo, 2014)?

ii) For how long can the embryos be maintained cryopreserved?

iii) What destinies should be available to surplus embryos (Goedeke *et al*., 2017): to postpone their cryopreservation until they naturally perish, to destroy them immediately, to use them for scientific purposes or to donate the embryos to third parties (a kind of prenatal adoption)? 

iv) Who is entitled to decide their destiny: the prospective parents (whether they are genetic parents or not), the ones that provided genetic material (disregarding if they intend to be legal parents or are mere donors), a representative of the embryos, the doctors, an independent authority or the courts?

v) Assuming that the decision belongs to the users of assisted reproductive technology, should the decision be taken in the informed consent form signed at the beginning of the reproductive treatment or only when the period of embryonic cryopreservation has expired? 

vi) If they are not required to make a decision beforehand and later they simply "disappear" or refuse to take a decision, what happens to the embryos?

vii) What if the prospective parents disagree regarding the embryos' destinies? (European Court of Human Rights, 2007)

In my opinion the users of reproductive techniques are the only ones entitled to decide the faith of surplus embryos, according with the options legally allowed, whether or not they are genetically connected with the embryos. They should be required to state their decision in the informed consent form, at the beginning of the reproductive procedure, although they can change their mind until the end of the cryopreservation period (to be established by law based on scientific findings). The decision should cover, not only the uses accepted for their surplus embryos, but also other scenarios, such as parental death or disagreement between the members of the couple. If the prospective parents refuse to take a decision or if they are in unresolvable conflict, the case should be decided by a court, having in consideration that embryos are not a human person nor they are entitled with rights, but still they are a form of human life that deserves respect.
